# Temporal specificity of the initial adaptive response in motor adaptation

**DOI:** 10.1371/journal.pcbi.1005438

**Published:** 2017-07-10

**Authors:** Wilsaan M. Joiner, Gary C. Sing, Maurice A. Smith

**Affiliations:** 1 John A. Paulson School of Engineering and Applied Sciences, Harvard University, Cambridge, Massachusetts, United States of America; 2 Sensorimotor Integration Laboratory, Department of Bioengineering, George Mason University, Fairfax, Virginia, United States of America; 3 Center for Brain Science, Harvard University, Cambridge, Massachusetts, United States of America; Johns Hopkins University, UNITED STATES

## Abstract

Repeated exposure to a novel physical environment eventually leads to a mature adaptive response whereby feedforward changes in motor output mirror both the amplitude and temporal structure of the environmental perturbations. However, adaptive responses at the earliest stages of learning have been found to be not only smaller, but systematically less specific in their temporal structure compared to later stages of learning. This observation has spawned a lively debate as to whether the temporal structure of the initial adaptive response is, in fact, stereotyped and non-specific. To settle this debate, we directly measured the adaptive responses to velocity-dependent and position-dependent force-field perturbations (vFFs and pFFs) at the earliest possible stage of motor learning in humans–after just a single-movement exposure. In line with previous work, we found these earliest stage adaptive responses to be more similar than the perturbations that induced them. However, the single-trial adaptive responses for vFF and pFF perturbations were clearly distinct, and the disparity between them reflected the difference between the temporal structure of the perturbations that drove them. Critically, we observed these differences between single-trial adaptive responses when vFF and pFF perturbations were randomly intermingled from one trial to the next within the same block, indicating perturbation response specificity at the single trial level. These findings demonstrate that the initial adaptive responses to physical perturbations are not stereotyped. Instead, the neural plasticity in sensorimotor areas is sensitive to the temporal structure of a movement perturbation even at the earliest stage in learning. This insight has direct implications for the development of computational models of early-stage motor adaptation and the evolution of this adaptive response with continued training.

## Author summary

With repeated exposure to a perturbation, the sensorimotor system learns to develop an adaptive response that is highly specific to both the amplitude and temporal structure of that perturbation in order to effectively counteract it. It is widely known that the amplitude of the adaptive response starts small and gradually grows to the right size with repeated exposure. However, it is also the case that the temporal structure of the adaptive response starts somewhat generically and gradually grows into the right shape with repeated exposure. A key question is whether the adaptive response to a perturbation begins with a stereotyped temporal structure that only becomes specified with further practice, or if it begins with a degree of specificity for the experienced perturbation that need only to be refined by practice. Here, by precisely measuring the temporal pattern of motor output in the single-trial adaptive response to two different perturbations, we show that the initial adaptive response is indeed specific to the temporal characteristics of the perturbation, even when the disturbance randomly changed from one trial to the next. These results demonstrate that the sensorimotor system is sensitive to the temporal features of a disturbance, even when experienced just once.

## Introduction

When voluntary movement encounters a physical perturbation, the motor system generates an adaptive response that counteracts the perturbation’s effects during subsequent movements. Several studies have suggested that this adaptive response is motion-state-dependent in the sense that it’s time course tends to be proportional to the time course of the position, velocity, and acceleration signals that characterize the motion. This is in line with the idea that motor adaptation acts to update an internal model of the physical environment which, based on Newtonian mechanics, should depend on motion state [1–9].

Several studies have shown that exposure to a perturbation can induce an adaptive response that is specifically tuned to the temporal structure of that perturbation [8,10–13] or a motion-state-dependent approximation of it [9]. This tuning can, however, be systematically biased. Sing et al. [8] found that that single trial exposure to pure position-dependent force-fields (pFFs) or pure velocity-dependent force-fields (vFFs) both induce adaptive responses with a partially velocity-dependent and partially position-dependent structure that gradually increase in specificity as adaptation proceeds. Due to this cross-adaptation, adaptive responses are not fully specific to the temporal structure of the perturbation. However, even at the earliest stage of learning, adaptive responses have been observed to be partially specific to the experienced perturbation, with the largest portion of the single-trial response being velocity-dependent for a vFF and position-dependent for a pFF [8,14]. Extended exposure to a particular FF environment further increases this specificity, and adaptive responses become highly specific after 60–100 exposures, even when washed out between exposures [14].

Two recent studies have reported, however, that single-trial adaptive responses display no specificity to the temporal structure of the perturbation. Fine and Thoroughman [15] examined brief force-impulse perturbations delivered at various points during movement, and Wei et al. [16] examined a variety of different perturbations including visuomotor rotation and linear and nonlinear position-dependent force-fields. Both reported identical single-trial adaptive responses to different perturbation types. This is grossly in line with the substantial cross-adaptation observed in Sing et al. [8] and Yousif and Diedrichsen [14], but at odds with the partial specificity observed in these same studies. Interestingly, the different perturbation types were randomly interleaved in the studies which found no specificity but blocked in the ones that did, leading Wei et al. [16] to suggest that the specificity observed in blocked experiments was due to a meta-learning effect arising from the multiplicity of single-trial exposures to the same perturbation rather than from any perturbation specificity inherent in the initial adaptive response.

As a counterpoint, both studies that reported non-specific responses largely based their findings on kinematic aftereffect data in which the temporal structure of adaptive response may have been obscured. This is the case because the effects of force adaptations would be filtered through the physical dynamics of the limb and combined with the effects of real-time feedback responses and limb impedance in producing aftereffect kinematics [17]. In contrast, Sing et al. [8] and Yousif and Diedrichsen [14] directly measured the temporal structure of the forces produced during the adaptive response using error clamp trials where no such filtering occurs and the effects of feedback control and limb impedance are minimized. Thus, it remains unclear whether the non-specific initial adaptation reported in the Fine and Thoroughman and Wei et al. studies resulted from better control of meta-learning-induced effects, or from lower fidelity kinematic measurements of the temporal structure of the adaptive response. Here, we address this question by directly measuring the forces produced during single-trial adaptive responses in an interleaved condition.

## Results

**Fig 1:**
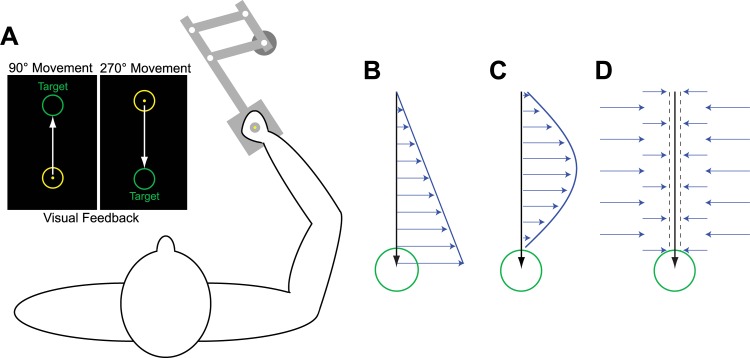
**(A) Experimental setup and trial types.** While sitting in front of a computer screen, subjects made reaching movements toward and away from the body (along the y axis) while holding the handle of the robotic manipulandum. Null trials were movements during which the motors of the manipulandum were turned off. There were three trial types during which the motors of the manipulandum were turned on. **(B)** During position-dependent force-field trials, the motors of the manipulandum produced forces on the hand (blue arrows) that were proportional in magnitude and perpendicular in direction to the position of hand motion (blue arrow). Forces (*f*) were calculated as a function of hand position: *f* = *Kx*. **(C)** During velocity-dependent force-field trials, the motors of the manipulandum produced forces on the hand (blue arrows) that were proportional in magnitude and perpendicular in direction to the velocity of hand motion (blue arrow). Forces were calculated as a function of hand velocity: $f=B\dot{x}$. **(D)** During error-clamp trials, the robot motors constrained the movements in a straight line toward the target by counteracting any motion perpendicular to the target direction.

**Fig 2:**
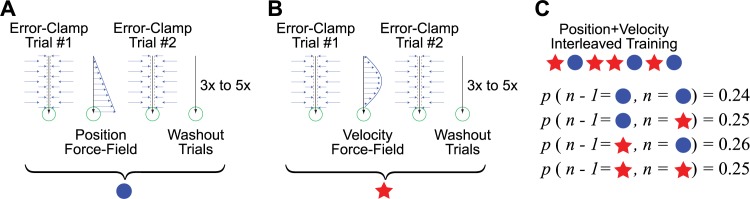
Experimental protocol. Single trial learning was examined by comparing the changes in lateral force between two successive error-clamp trials separated by a force-field (FF) trial. These three trials formed a EC-FF-EC measurement triplet. Experiments were based on triplets built around position-dependent FF trials (pFFs) and velocity-dependent FF trials (vFFs). **(A)** a position-dependent force-field flanked by two error-clamp trials and **(B)** a velocity-dependent force-field flanked by two error-clamp trials. Consecutive EC-FF-EC triplets in each movement direction were separated by 3–5 null-field trials. **(C)** In the experiment, triplets were randomly ordered and balanced across FF type (pFF vs vFF), FF direction (clockwise vs counter-clockwise), and movement direction (90° vs 270°).

In this study we examined the adaptive changes in motor output at the earliest stage in learning–after a single trial–in order to determine the relationship between the temporal structure of the adaptive response and the temporal structure of perturbations we imposed to drive this adaptive response. We focused on two types of dynamic perturbations: velocity-dependent and position-dependent force-fields (vFFs and pFFs) that were applied to point-to-point reaching movements, as illustrated in Fig 1. In particular, we examined single-trial adaptive responses (Fig 2A and 2B) when these two FF perturbations were randomly interleaved (Fig 2C). We measured these responses by flanking each vFF or pFF perturbation trial with two error-clamp (EC) trials (Fig 2A and 2B), so that we could take the difference between the lateral force profiles measured before and after FF exposure (see Methods). To prevent adaptation from building up during the experimental session so that the adaptive response to each perturbation could be measured independently, we inserted 3–5 null-field washout trials between each EC-FF-EC triplet and randomized the sign (direction) of the FF from one exposure to the next.

### Kinematics of the force field perturbations

**Fig 3:**
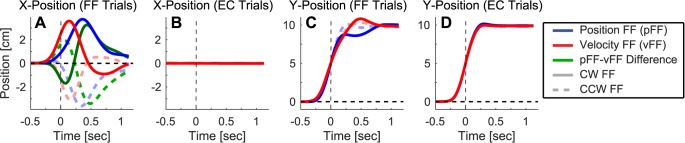
Hand displacement during velocity-dependent and position-dependent single trial learning. The average perpendicular (X-axis) displacement of the hand path (along the x-axis) as a function of time for **(A)** force-field (FF) and **(B)** error-clamp (EC) trials. The blue and red traces represent position-dependent force-field (pFF) and velocity-dependent force-field (vFF) perturbations respectively. The green traces represent the difference between the blue and red traces. Clockwise (CW) FF data are illustrated with bright solid lines, and counter-clockwise (CW) FF data are illustrated with lighter dashed lines. Longitudinal (Y-axis) hand paths are shown as a function of time for **(C)** FF and **(D)** EC trials. Zero ms in all panels corresponds to the midpoint of the movement.

We found that the pFF and the vFF perturbed movements by similar amounts, with 3–4 cm maximum displacements in all cases (Fig 3A). Note that 0 ms in Fig 3A–3D corresponds to the midpoint of the movement. The vFF produced more lateral displacement early in the movement but less displacement late than the pFF (Fig 3A). This is consistent with the fact that the perturbing force in the vFF is larger in amplitude than in the pFF early in the movement but smaller late in the movement. Correspondingly, the peak displacement occurred significantly earlier for velocity-dependent FF perturbations (155 ± 5 ms (mean ± SEM) vs. 339 ± 10 ms for clockwise FFs and 140 ± 7 ms vs. 352 ± 12 ms for counter-clockwise FFs, *p* < 10^−6^ in both cases), in line with greater early-movement effects for vFF perturbations and greater late-movement effects for pFF perturbations. Fig 3B shows that the lateral displacements in EC trials were kept extremely small, generally less than 0.6 mm.

We found longitudinal motion (Fig 3C) to be considerably less affected than lateral motion for both the pFF and the vFF perturbations, in line with the fact that both were curl FFs that produced forces in a direction orthogonal to motion (see Methods). Correspondingly, the correlation coefficients between the pFF and vFF perturbation trial data were substantially higher for longitudinal position data (0.997 for CW fields, 0.985 for CCW fields, Fig 3C) than for lateral position data (0.310 for CW fields, 0.321 for CCW fields, Fig 3A). For reference, we found correlations of 1.000 for both analogous comparisons for the longitudinal position data in the EC trials following each perturbation type (Fig 3D).

### Single-trial adaptive responses for position- and velocity-dependent perturbations

**Fig 4:**
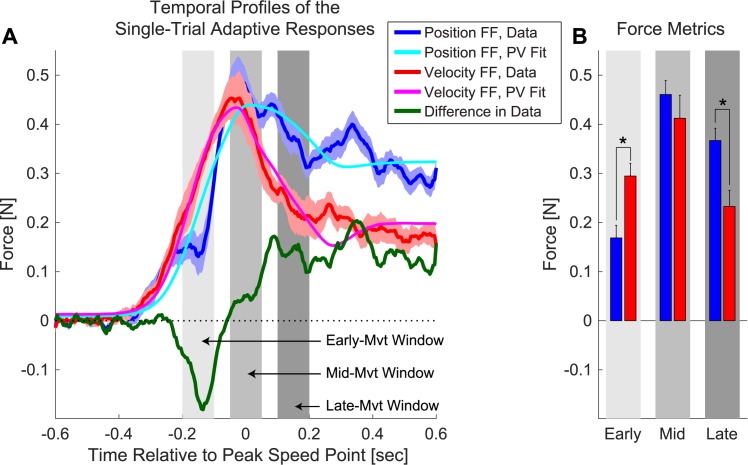
Force profiles during velocity-dependent and position-dependent single trial learning. **(A)** Force profiles showing the average temporal structure of the adaptive response for pFF (blue) and vFF (red) trials. Background shading shows 1-SEM error bars. Lighter blue and pink traces represent motion-dependent fits to the force profiles for pFF and vFF perturbations, respectively. The respective motion-dependent fits were determined by simultaneously regressing each baseline-subtracted force pattern onto a linear combination of the position, velocity, and acceleration profiles associated with movement. The thick green trace represents the difference in perpendicular force between the pFF and vFF adaptive responses. That this trace crosses zero, indicates that pFF responses are sometimes larger and sometimes smaller than vFF responses which can only occur if these responses have distinct shapes. **(B)** Comparison of the adaptive responses for pFF and vFF perturbations at early, middle and late points in the movement, based on the 100ms-wide windows shown in panel A. Asterisks represent significant differences in force between pFF and vFF responses at (*p* < 0.01, two-tailed t-test). Error bars show SEM.

The single-trial adaptive responses displayed in Fig 4A show that pFFs and vFFs induce adaptive responses with different temporal structures. Note that 0 ms in Fig 4A corresponds to the midpoint of the movement. Although the longitudinal motion profiles associated with the pFF and the vFF data are essentially identical (Fig 3D), and the peak force levels during the adaptive responses are quite similar (0.45–0.5N, see Fig 4A), the shapes of the force profiles that characterize these responses appear systematically different. The pFF response (blue) is smaller early in the movement and larger late in the movement than the vFF response (red), as illustrated by the thick green trace representing the difference between the pFF and vFF responses in Fig 4A. Grossly, this response pattern echoes the temporal pattern of errors induced by the perturbation (Fig 3A). The larger early-movement vFF response is consistent with the larger early-movement vFF perturbation effect shown in Fig 3A, and the larger late-movement pFF response is consistent with the larger late-movement pFF perturbation effect shown in Fig 3A.

To quantify differences between the temporal structure of the single-trial adaptive response to pFFs and vFFs, we analyzed the force data from three distinct 100ms windows: centered at the mid-movement point (mid), 150 ms before this point (early), or 150 after it (late). We operationally defined the mid-movement point as the time point where the longitudinal (Y) component of the position profile crossed the 5 cm point of the 10 cm-long movement. Analysis of the adaptive responses during these three windows reveals the pFF response to be 43 ± 12% *smaller* than the vFF response in the early-movement window, but 58 ± 13% *larger* than the vFF response in the late-movement window as illustrated in Fig 4B (*p* < 0.008 and *p* < 0.002, respectively, two-tailed paired t-tests). The adaptive responses are nearly equal during the mid-movement window, with a nominal difference of 12 ± 8%, *p* = 0.19. These data indicate that the time course of the adaptive response is not stereotyped as previously suggested [15,16].

### Gain-space analysis of single-trial adaptive responses

**Fig 5:**
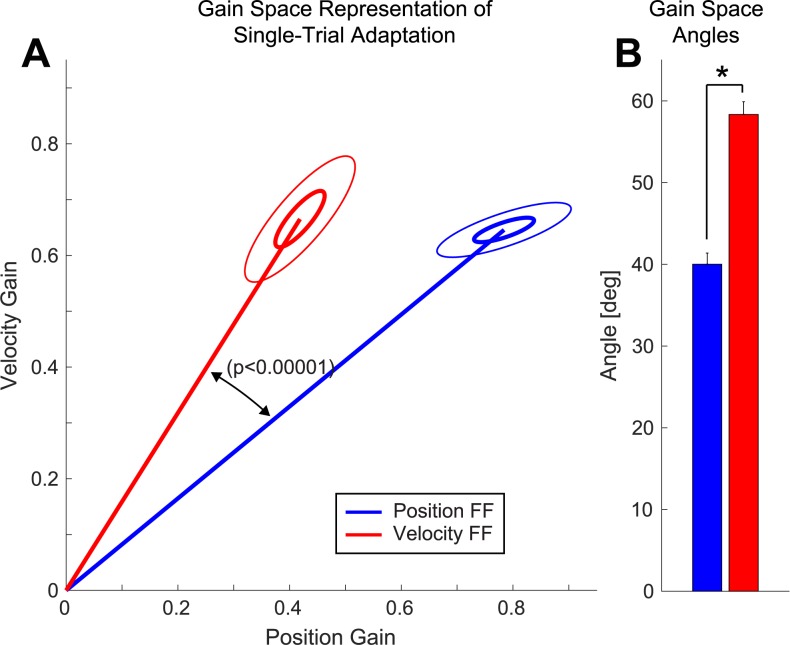
Gain-space representation of velocity and position contributions to lateral force for single trial learning. **(A)** Gain space representations for single-trial pFF (blue) and vFF (red) adaptation. Ellipses represent 1-SEM (inner) and 95% confidence interval (outer) error bars in the position-velocity (PV) gain-space. **(B)** Average angle in PV gain space for single-trial pFF and vFF adaptive responses. Symbols represent a significant difference in PV gain-space angles between pFF and vFF adaptations (*p* < 0.01, two-tailed t-test). Error bars show SEM.

We previously found that adaptive responses to position-dependent and velocity-dependent force-fields were well-characterized by a linear combination of motion-dependent responses [8]. This is shown for the current data set in Fig 4A. Analyzing adaptive responses in the space of position and velocity gains (the PV gain-space, see methods) can provide a compact, low-dimensional (two-parameter) characterization of the shape of each adaptive response and allow a direct visualization of its specificity, as shown in Fig 5. More specifically, this PV gain-space analysis directly relates the temporal structure of the adaptive responses to the temporal structures of the position-dependent and velocity-dependent perturbations that induced these responses. A vFF perturbation would induce a purely velocity-dependent response if perfect specificity were maintained, leading to an adaptation vector in PV gain-space with only a velocity-dependent component. This would be represented by an adaptation vector in Fig 5A perfectly aligned with the (vertical) velocity axis of the PV gain-space. Likewise, a perfectly specific response to a pFF perturbation would be represented by an adaptation vector perfectly aligned with the (horizontal) position axis of the PV gain-space.

Consistent with the direct analysis of the adaptive response profiles shown in Fig 4, the gain-space plots in Fig 5A show that the position- and velocity-dependent force-field perturbations induce adaptive responses with distinct shapes. We find that the vFF response vector is more strongly velocity-dependent (i.e. closer to the y-axis) than the pFF response which is more strongly-position dependent (i.e. closer to the x-axis). Here the difference in the temporal structure of the response shape can be characterized, independent of response amplitude, by the angle in gain-space between the pFF and vFF responses. Note that gain-space angle (*θ*) for a particular response is a monotonic function of the velocity-position gain ratio: *θ* = *arctan*(*G*_*V*_ /*G*_*P*_) where *G*_*P*_ and *G*_*V*_ are the position and velocity components of the gain-space response. Analysis of the gain-space angles for pFF and vFF responses reveals that the vFF responses are consistently greater in angle (with respect to the x-axis) than the pFF responses as shown in 5B (Δ*θ* = 18.3° ± 1.9°, *p* <10^−5^). This indicates that although the adaptive responses to the pFF and vFF perturbations are far from perfectly specific, they are clearly distinct from one another. This finding corroborates the movement segment analysis illustrated in Fig 4, which also finds differences in the pFF and vFF responses, and shows that the differences we observe in these adaptive responses directly correspond to response specificity: pFF perturbations elicit adaptive responses with a relatively greater position component leading to systematically smaller gain-space angles across participants, whereas vFF perturbations elicit adaptive responses with a relatively greater velocity component leading to systematically larger gain-space angles. Note that the gain space analysis presented in Fig 5 is intrinsically more powerful than the early and late movement segment comparisons presented in Fig 4, because it considers the entire time course of the adaptive response.

The randomly interleaved exposure to pFF and vFF perturbations in the current experimental paradigm (Fig 2C) was put in place so that meta-learning about the perturbation type would be minimized. Thus, the current results indicate an inherent temporal specificity to the adaptive response that cannot be due to meta-learning. However, it might be that repeated exposure to the same perturbation type might lead to meta-learning that further increases response specificity beyond that observed in our interleaved data. To examine this possibility, we compared the current results to a previous dataset [8] with a similar experimental design, but where only a single perturbation type was experienced by each participant. Like the current experimental design, single-trial adaptive responses were measured following occasional curl pFF and vFF perturbations with a probability of 0.2 and the direction of the perturbations was randomly interleaved; however, one group of participants experienced only vFF perturbations and a second group only pFF perturbations.

We compared the gain-space representation of the single trial learning (described in Fig 5) between the two experimental designs: single-field versus interleaved. We found that neither the angles nor amplitudes of the gain-space vectors were different between pFF single-field versus pFF interleaved data (*p* > 0.2 in both cases) or for the vFF single-field versus vFF interleaved data (*p* > 0.3 in both cases). In particular, the adaptive response specificity as assessed by the difference in PV gain-space angles between vFF and pFF perturbations was similar for the single-field (previous) and interleaved (current) data (22.8° ± 4.7° versus 18.3° ± 1.9°, *p* > 0.3). This data cannot rule out the possibility that meta-learning contributed to the adaptive response specificity observed in Sing et al. [8], however it appears that meta-learning plays a substantially smaller roll, if any, than the inherent adaptive response specificity that we elucidate in the current study, suggesting that meta-learning effects are small for the isolated single-trial adaptation paradigm studied in Sing et al. [8]. Interestingly, we recently found large meta-learning effects for the amplitude of the adaptive response for training paradigms with high environmental consistency in highly-structured environments where multiple FF trials consecutively encountered [12]. It would be interesting to determine where such environment could also increase the temporal specificity of the adaptive response.

## Discussion

We studied the adaptive changes in motor output during motor learning and found distinct temporal structure in the adaptive responses to two different force-field perturbations, indicating that initial adaptive responses are not stereotyped. The adaptive response to a position-dependent force-field (pFF) was significantly smaller than the velocity-dependent force-field (vFF) response early in the movement, whereas the pFF response was significantly larger than the vFF response late in the movement (Fig 4). The observed pattern is in line with the fact that for the point-to-point reaching movements we studied, velocity-dependent forces peak earlier and position-dependent forces peak later. This suggests that single-trial adaptive responses are not only demonstrably different in shape, but that the observed difference reflects the shapes of force patterns associated with the position-dependent and velocity-dependent force perturbations that induced the adaptive responses. A motion-dependent regression analysis confirmed this possibility, showing the adaptive response to a single vFF exposure to have a significantly greater velocity-position ratio than the adaptive response to a single pFF trial (Fig 5). Critically, the experimental paradigm was designed so that pFF and vFF perturbations were randomly interleaved, minimizing potential meta-learning effects. Taken together, these data provide clear evidence for distinctly different adaptive responses that display clear inherent specificity for different types of physical dynamics at the earliest possible point of motor adaptation—after just a single exposure to a perturbation.

### Physiological implications of early learning responses that display position-velocity cross-adaptation

Although we have clearly demonstrated that adaptive responses to position-dependent and velocity-dependent force-field perturbations are distinct even after a single trial of adaptation, these single-trial responses are far from fully specific. We found that the difference in gain-space angles between the pFF and vFF responses to be 18.2 ± 1.9°– just over 20% of the 90° angle that would be expected for fully specific responses. Correspondingly, the correlation coefficient between the average pFF and vFF responses shown in Fig 4A is *r* = 0.84, whereas the correlation coefficient between the shapes of the perturbations themselves was *r* = 0.02.

Correlated, but distinct adaptive responses for pFF and vFF perturbations, like those that we observe here, are consistent with motor primitives that display correlated tuning to the position and velocity of movement [8,9,13]. Correlated tuning to these motion states is observed throughout the sensorimotor system; motor spindle afferents [18,19], neurons in premotor and primary motor cortex [20–22], and the cerebellum [23] demonstrate codependent encoding of position and velocity during movement. There is increasing evidence that the motor system represents force perturbations to movement based on this tuning, and that this correlated representation of motion states influences the observed adaptive response. For example, in a previous study we showed that adaptive responses to single-trial force-impulse perturbations were highly motion dependent, although the dynamics of the perturbation was not [9]. This common tuning to position and velocity is also in line with the current results, insofar as we observe substantial cross-adaptation in response to position-dependent and velocity-dependent dynamics. We find the degree of cross-adaptation to be, however, incomplete, resulting in initial adaptive responses that are only partially stereotyped. And following extended exposure, adaptive responses gradually become more specific—revealing that the underlying neural networks are modified with training to better reflect the temporal structure of environmental dynamics [8,9]. Importantly, the specificity observed following single-trial exposure in the current study suggests that neural plasticity in sensorimotor areas reflect the temporal structure of the perturbation.

### Previous work on the temporal specificity of the initial adaptive response

In previous work [24] we showed that the pattern of generalization of the adaptive response in early learning matched that predicted by the actual rather than the planned motion (motion-referenced learning) during training. That is, when the movement goal was manipulated such that subsequent movements were aligned to the actual motion experienced during training (the perturbed trajectory) there was a significant improvement in the learning rate, and a significant reduction when there was a misalignment of the two, in line with the idea of motion-referenced learning. Further evidence for motion-dependent learning comes from a recent study in which adaptive responses to perturbations that were themselves not motion-dependent [9]. In this study, two different narrow force-impulse perturbations that could not be well-approximated by a motion-dependent representation comprised of a linear combination of position, velocity, and acceleration signals, induced adaptive responses that were consistently well-explained by such a motion-dependent representation. The study found this to be the case, both for single-trial adaptation and after extended exposure. As the duration of the training exposure increased, the internal composition of the motion-dependent representation evolved substantially but the overall degree of motion dependence remained. Together with the current results, these findings paint a picture of the initial adaptive response that is strongly motion-dependent and flexible enough within the space of motion-dependent representations to display a clear amount of inherent specificity to the temporal structure of the experienced perturbation.

The current results for interleaved training are consistent with our previous findings for single-field training during which the FF type was held constant throughout the experiment [8]. In that study the direction of the perturbation was also changed randomly throughout the session with a short washout period between the error-clamp force-field measurement triplets. Before the current study, it could be argued that these prior results for single-field training resulted primarily from some form of meta-learning [16], whereby the temporal structure of the adaptive response on any particular trial is fixed but can be gradually reshaped by repeated exposure to a particular perturbation. The idea was that meta-learning that involves gradual reshaping the temporal structure of the adaptive response over the course of training might be responsible for the adaptation specificity observed in non-interleaved training paradigms. However, in the current study we find that the same pattern of specificity is maintained when the perturbation types are interleaved so that the initial adaptive responses to different perturbations are measured in rapid succession. This indicates the adaptive responses are inherently specific at the earliest stage in the adaptive response. Moreover, when we examined whether meta-learning might further increase this inherent specificity, we found little effect: With our most powerful measure of response specificity (the difference in PV gain-space angles between pFF and vFF responses), we found no significant increase for single-field learning where meta-learning might be able to contribute, and the nominal increase in this measure was only 1/5 as large as the inherent specificity we observed, suggesting that the inherent specificity of the initial adaptive response can dominate meta-learning driven effects.

However, the current results appear grossly at odds with the findings from two previous studies that were unable to find evidence for temporal specificity in the initial adaptive response. In the first study by Fine and Thoroughman [15], subjects occasionally experienced a single force pulse perturbation during point-to-point reaching movements. The force pulses were applied orthogonal to the movement direction, but the direction, magnitude and location within the movement the pulse was applied varied from trial to trial. The authors observed that the adaptive response, measured in the null aftereffect trials immediately after these force pulse movements, was sensitive to the direction, but insensitive to the amplitude and the time within the movement the force pulse was applied. However, the kinematic assessment of adaptation aftereffects with null trials is sensitive to the online feedback correction and the changes in limb impedance that dominate compensation late in the movement [3,7,17,25–30]. This obscures late-movement feedforward changes in adaptive control, which reduces the ability to accurately determine the temporal structure of the feedforward adaptive response. A key feature of the current study is the use of error-clamp trials to probe the dynamics of the feedforward adaptive changes in force output throughout the entire movement [8,31,32]. We show that the adaptive response to each force field continues throughout the course of the movement (see Fig 4), and that information about the entire time course–allowing the comparison of early and late forces during each movement–is required to demonstrate the temporal specificity we report. Compared to pFF perturbations, vFF perturbations result in an increased early-movement adaptive response alongside a decreased late-movement adaptive response. Indeed, it is the coexistence of these opposing early- and late-movement differences that indicates the specificity of the temporal structures of the adaptive responses to pFF and vFF perturbations. Consistent changes in the adaptive response early, mid, and late in movement could result from a gain change alone. However, opposing differences in the adaptive response at distinct time points such as early and late into the movement is strong evidence that the temporal structure of the adaptive response is specific to different perturbations.

Recently, Wei et al. also looked at responses to various force perturbations during point-to-point reaching movements implemented as different functions of longitudinal position [16]. Although they characterized the set of perturbations they studied as “random”, four of the five different force perturbations they used closely resembled or exactly matched, linear functions of motion-state. In particular, their “ramp” perturbation was exactly linearly position-dependent, their “half-sine” and “triangle” perturbations were almost linearly velocity-dependent (correlation coefficients > 0.92 if a bell-shaped minimum-jerk velocity profile is assumed in both cases), and their “sine” perturbation is almost linearly acceleration-dependent (correlation coefficient > 0.85). Thus, 3 of the 5 force perturbations employed in Wei et al. [16] were extremely similar to the position- and velocity-dependent dynamics examined in the current study, which should make much of the data highly comparable.

In their first experiment, the authors looked at the motion aftereffects induced by these perturbations, which, as argued above are unlikely to give substantial insight into the time-course of the feedforward adaptive response. In their second experiment, Wei et al. [16] analyzed the adaptive responses observed on error-clamp trials following the application of these perturbations, although details of the experimental design and analysis make interpretation of the results, unfortunately, rather difficult. A key issue is that the experiments attempted to look at single-trial learning by examining aftereffects following perturbations that were densely spaced. Perturbations were levied on 50% of trials, some but not all of which were followed by aftereffect trials used to estimate the single-trial adaptive response. Critically, no attempt was made to wash out learning from one perturbation trial to the next, allowing the adaptive response to display a random-walk-like buildup across different perturbation types during the experiment. This buildup would preclude a clean interpretation of the aftereffect responses observed following each different perturbation type because the adaptive response following an individual perturbation trial would partly result from that perturbation itself and partly from perturbations that closely preceded the trial of interest. Due to the random 50% perturbation rate, one would, in fact, expect the trial immediately preceding a perturbation to itself include a perturbation in 50% of cases, and the two immediately preceding trials to both include perturbations in 25% of cases. Contamination from preceding trials with different perturbations might have been mitigated if the experiment had included probe trials both before and after each perturbation so that the learning-related change in the adaptive response could be estimated from the difference in the corresponding aftereffects to subtract out previous learning. However, such trials were not included, and no attempt to subtract out the buildup of adaptation from exposure to preceding perturbations was made. Both the current study and Fine and Thoroughman [15] included a number of washout trials between perturbations as well as probe trials before and after perturbations to mitigate the non-specific buildup mentioned above. Keeping non-specific effects at bay is critical when examining the specificity of the adaptive response. It is therefore unclear whether the Wei et al. findings were driven by the non-specific nature of the experimental design and analysis performed.

### The amplitude specificity of the initial adaptive response

In addition to temporal specificity, Fine and Thoroughman [15] also examined the amplitude specificity of the initial adaptive response by looking at single-trial adaptation following different perturbation amplitudes. They found that although brief force-pulse perturbations of 6N, 12N and 18N, induced motor errors in proportion to perturbation amplitude, the adaptive responses measured by the post-perturbation after-effect size were similar to one another. The authors interpreted this as evidence for a fixed-amplitude for the initial adaptive response.

However, the data from the individual conditions presented in Fig 4 of the Fine and Thoroughman study reveal the 12N perturbation responses to be nominally larger that the 6N responses in 5 of 6 individual conditions examined, and the combined data presented in Fig 5 of that paper indicate a nominal increase in adaptive response strength of 30–50% in the 12N responses compared to the 6N responses. Both suggest that perturbation response amplitude may increase with perturbation amplitude. It should be noted, however, that 100% increases would be expected for a linear increase in perturbation response amplitude from 6N to 12N, and that no consistent increases in perturbation response strength were evident in going from 12N to 18N perturbations. Thus, the Fine and Thoroughman [15] data clearly demonstrate that the relationship between perturbation amplitude and adaptive response amplitude is sublinear. However, the claim of a fixed-amplitude single-trial adaptive response is unclear, as the data appear to suggest a relationship that increases for smaller perturbation amplitudes, in line with a saturating non-stereotyped adaptive response amplitude. Four more recent studies are also in line with a sublinear but non-stereotyped adaptive response amplitude for single-trial adaptation to both force [33,34] and visuomotor [35,36] perturbations. In these studies, initial adaptive responses systematically increased in amplitude with the amplitude of lateral perturbations, but the rate of the observed increased displayed a progressive reduction and then leveled off for large perturbation amplitudes. Taken together, these studies show that the amplitude of single-trial adaptive responses are not stereotyped, but systematically depend on perturbation amplitude. This indicates that adaptive responses are at least partially specific to the amplitude of environmental perturbations, consistent with the current findings that adaptive responses are specific to the temporal structure of environmental perturbations.

### Conclusion

In this study we have shown that single-trial learning of position- and velocity-dependent force-fields leads to distinct adaptive responses, contradicting the claim that initial adaptation to unexpected or random perturbations is stereotyped. This was true when the two perturbation types were interleaved throughout the training session—preventing effects due to meta-learning from constant exposure to a single perturbation. When we examined the effect of meta-learning from exposure to a single perturbation by comparing the adaptive responses from single-field vs interleaved conditions, there was little to no effect. There was no significant improvement in response specificity. Together these results suggest that even after a single perturbation exposure, the motor system is sensitive to the characteristics of the disturbance to movement and utilizes this information in the compensatory motor output.

## Materials and methods

### Participants

Eleven healthy subjects without known neurological impairment were recruited from the Harvard University community to participate in the study. All participants were right handed and performed the task using their right hand. The study protocol was approved by the Harvard University Institutional Review Board and all participants gave informed consent.

### Experimental setup

The experimental paradigm was based on the standard force-field (FF) adaptation paradigm [1]. Subjects were trained to move their hands to targets in the horizontal plane while grasping a robot manipulandum (Fig 1A). The manipulandum measured hand position, velocity, and force, and its motors were used to apply forces to the hand, all at a sampling rate of 200 Hz. The position of the hand was shown as a small round cursor (3 mm) on a vertically oriented computer monitor in front of the participant (refresh rate of 75 Hz). Participants reached to circular targets 1 cm in diameter that were spaced 10 cm apart. We instructed participants to ‘‘make quick movements to the targets.” In addition, subjects were told that the reaction time was not important—they could wait as long as they wished after target appearance before starting each movement—but when ready, they were to move in a rapid motion toward each target. The endpoint of each movement was used as the starting point for the subsequent movement, and movements were made in two target directions [37].

Three trial types were used in the experiment: null field trials, force field (clockwise or counterclockwise) trials, and error-clamp trials (Fig 1B, 1C and 1D). Null field trials (or simply, null trials) were used for initial familiarization with the experiment and for washout between force field trial exposures in order to minimize the buildup of adaptive responses from one force field exposure to the next (see below). During these trials the motors of the robot manipulandum were turned off. The second type of trial was a force field trial. We studied two types of force-field trials with different temporal structures so that the temporal specificity of the adaptive response could be assessed. During position-dependent force-field (pFF) trials (Fig 1B) the relationship between force (*f*) and position (*x*) vectors was determined by the 2x2 matrix: *K* = [0 *δ*;−*δ* 0] via the relationship *f* = *Kx*, with *δ* = ±45 *N*/*m* so that the forces imposed on the hand were proportional in magnitude and perpendicular in direction to hand displacement along the target axis. For velocity-dependent force-field (vFF) trials, the motors were used to produce forces on the hand that were proportional in magnitude and perpendicular in direction to the velocity of hand motion (Fig 1C). In this case, the relationship between force (*f*) and velocity ($\dot{x}$) vectors was determined by the 2x2 matrix: *B* = [0 *α*;−*α* 0] via the relationship $f=B\dot{x}$, with *α* = ±15 *N*/(*m*/*s*). The direction of force-field (clockwise or counterclockwise) changes when the sign of *α* (for velocity force-fields) or *δ* (for position force-fields) is changed. The third type of trial was an error-clamp (EC) trial. During EC trials, the robot motors were used to constrain movements in a straight line toward the target by counteracting any motion perpendicular to the target direction [7–9,11–14,24,31,32,34,37–41] in order to clamp lateral displacements and thus lateral errors to near-zero values. In these trials, perpendicular displacement from a straight line to the target was generally held to less than 0.6 mm and averaged about 0.2 mm in magnitude. This was achieved by applying a stiff one-dimensional spring (6 kN/m) and damper (150 Ns/m) in the axis perpendicular to the target direction.

### Task

Subjects made repeated point-to-point movements between two target positions that were both in the participant’s midline. The movement thus alternated between outward movements (that we refer to as 90° movements) and backward movements (that we refer to as 270° movements). The experiment began with a 270-trial familiarization period (135 trials in each movement direction) that did not include FF trials and was divided into 3 blocks separated by short one-minute rest breaks.

The main experiment period followed in which we studied the specificity of initial motor adaptation by examining adaptation after just a single exposure to either a pFF or vFF perturbation. To accomplish this, each FF trial was flanked by EC trials in the same movement direction (thus each flanking EC trial was 2 trials away from the middle FF trial) forming an EC-FF-EC measurement triplet. These EC trials provide the ability to accurately measure the temporal pattern of lateral forces associated with each movement [8]. Thus the measurement triplets provide the ability to accurately measure the temporal structure of the adaptive response to the laterally-directed pFF and vFF perturbations we studied by examining the difference in the lateral force profiles observed before vs after pFF and vFF perturbations. This post-FF minus pre-FF difference was the main experimental result that we analyzed (see below).

To prevent adaptive responses from building up from one triplet to the next, we followed each triplet with 3–5 null field trials in that triplet’s movement direction and randomly ordered the direction of the FF perturbations experienced in consecutive triplets (clockwise vs counter-clockwise). FF exposures only occurred within measurement triplets and the 224 exposures in the experiment were balanced across movement direction (90° vs 270°), perturbation direction (clockwise vs counter-clockwise), and perturbation type (pFF vs vFF) and presented in a randomized order.

A critical feature of our experimental design was that pFF and vFF perturbations were randomly interleaved (Fig 2C) so that meta-learning about the perturbation type to be expected would be minimal. Furthermore any meta-learning that might occur could not systematically effect measurements of the adaptive response to pFF vs vFF perturbations (because the type of perturbation in any given triplet would be independent of the perturbation type previously experienced).

### Data analysis

Based on the experimental design, the differences between the force profiles measured on the post-FF versus pre-FF trials from the measurement triplets for pFF and vFF perturbations was the main focus of the analysis. These difference are plotted for the pFF and vFF data in Fig 4A and analyzed in Figs 4 and 5. We combined the data from CW and CCW perturbation directions, aligning the force profiles to the perturbation, and we additionally combined the 90° and 270° movement data. In general, lateral force could reflect an adaptive compensation of expected external force or an online feedback correction for errors detected during the course of movement. However, on the EC trials on which the lateral force were measured, lateral errors were small so that online feedback correction should have little effect. Thus the lateral forces measured on EC trial should primarily reflect feedforward motor adaptation.

One of the eleven participants did not consistently remain on-task in terms of making the rapid movements instructed. We thus excluded his data. The participant’s movements were nearly 5 standard deviations slower than the mean of the other 10 participants. For the remaining data we excluded the small fraction of trials (2%) that had very slow or very fast movement speeds (peak speed < 0.22 m/s or > 0.50 m/s) or very high peak force levels (>15 N). Since the number of washout trials (3–5) between the triplets in the 90° and the 270° directions were randomized independently, 90° perturbations were presented in an order that was entirely independent of the 270° perturbations. Thus FF perturbations on 90° and 270° trials sometimes occurred several movements apart and sometimes occurred directly adjacent on another. The latter circumstance resulted in some “shared” triplets–triplets in which a perturbation trial in the 270° direction occurred adjacent a 90° perturbation trial and thus between the 90° pre-FF and post-FF EC trials, or vice versa. When we examined these shared triplets, we found that shared triplets in which perturbations were both in the same direction (either both CW or both CCW) appeared to show somewhat different pre-FF / post-FF changes than shared triplets in which perturbations were in opposite directions (possibly providing interfering vs facilitating effects), even though the 90° and 270° movement directions were separated by 180°—a large difference in movement directions over which generalization in often minimal. To avoid the possibility that interactions between movement directions might play a role in the shaping of the single-trial adaptive responses we report, we included only the data from “solitary” triplets in our analysis of the force profile data illustrated in Fig 4A and the subsequent analyses based on these force profiles which follow. These solitary triplets comprise 58% of the measurement data, and the shared triplets comprise 42%. Including just the solitary triplet data versus the entire set of the triplet data turns out to have little systematic effect on the results as shown in S1 Fig plotted below, which reveals that systematic differences in the temporal structure of pFF vs vFF adaptive responses are maintained for both the all-triplet data and the solitary-triplet-only data. The most noticeable difference between these two is that the 42% smaller data solitary-triplet-only dataset appears noticeably noisier in shape than the entire data set, as would be expected given the smaller size. Regardless, we thought it might be best here to be methodologically cautious and include only the cleanly-designed solitary-triplet data.

We took the approach of assessing the specificity of the temporal structure of the force profiles associated with single-trial adaptation by (1) analyzing whether pFF and vFF perturbations induced adaptive responses with distinct temporal structure and (2) examining whether the difference in the temporal structure of the adaptive response for pFF vs vFF perturbations reflected the differences in the perturbations themselves. We performed two different analyses, each of which could shed light on both of these issues. The first analysis, illustrated in Fig 4, simply examined the time course of the adaptive responses to pFF vs vFF perturbations by comparing these adaptive responses at three time points: early, middle, and late in movement. Specifically, we computed the mean force during 100ms windows centered 150ms before the middle of the movement, at the middle, and 150 ms after the middle. Thus the early period ranged from -200 to -100 ms with respect to the mid-movement point, the mid period ranged from -50 to 50 ms, and the late period ranged from 100 to 200 ms. We operationally defined the mid-movement point as the time when the longitudinal position crossed 5 cm of the 10 cm-long movement. Distinct temporal structure would be indicated by differential findings at early vs mid vs late-movement time points such as significantly decreased lateral force at one time point alongside significantly increased force at another. More specifically, temporal specificity in the adaptive response would predict that pFF responses would be significantly decreased when compared to vFF responses early in the movement yet significantly increased when compared to vFF responses late in the movement because pFF perturbations peak later than vFF perturbations.

The second analysis, illustrated in Fig 5, directly assessed the position-dependence and velocity-dependence of the pFF and vFF adaptive responses, in line with the idea that temporal specificity in the adaptive response would predict that pFF responses should be more strongly position-dependent and vFF responses should be more strongly velocity-dependent when compared to one another. We performed this analysis by projecting the pFF and vFF adaptive responses into a position-velocity gain-space (PV gain-space). This was accomplished by first regressing the experimentally measured force profiles onto a motion-dependent representation consisting of the position, velocity, and acceleration of movement as well as a constant offset, and then determining the gains (*G*_*P*_ and *G*_*V*_) associated with the position-dependent and velocity-dependent regression coefficients (*C*_*P*_ and *C*_*V*_) compared to the strengths of the pFF and vFF perturbations (*δ* & *α*). Thus, *G*_*P*_ = *C*_*P*_ / *δ* and *G*_*V*_ = *C*_*V*_ / *α*. Note that this procedure is analogous to simultaneously computing adaptation coefficients [8,32,38,39] for both the pFF and VFF perturbations for each type of adaptive response. If the measured lateral force and the ideal force for one FF were to perfectly coincide (i.e., full FF compensation), the corresponding gain from the linear regression would be 1, if the two profiles were directly opposed, the gain would be -1, and if they were unrelated it would be zero. Note that we used a motion-dependent representation that contained acceleration because it has been shown to provide an efficient low-dimensional representation of the temporal structure of the adaptive response to a variety of different force perturbations–for multiple motion-dependent perturbations and force impulse perturbations [8,9]. In particular, this removes contamination of the PV gains by a significant acceleration-dependent response (and note that pure PV projection also yields result of even greater amplitude and as statistically powerful as the more refined gain-space analysis we employ). The PV gain-space representations we computed in Fig 5 are summarized for statistical testing by the direction (angle) of the PV gain-space vector for each FF.

## Supporting information

S1 Fig Force profiles during velocity-dependent and position-dependent single trial learning for different portions of the data set. Layout is the same as Fig 4. Force profiles showing the average temporal structure of the adaptive response for pFF (blue) and vFF (red) trials for **(A)** the complete data set (a combination of the “shared” and “solitary” triplets, see Materials and Methods for details) and **(C)** the “solitary” only triplets. In each panel background shading shows 1-SEM error bars. Lighter blue and pink traces represent motion-dependent fits to the force profiles for pFF and vFF perturbations, respectively. The thick green trace represents the difference in perpendicular force between the pFF and vFF adaptive responses. Respective comparison of the adaptive responses for **(B)** the complete data set and **(D)** the “solitary” only triplets. In each panel the magnitude of the force profile response for pFF and vFF perturbations at early, middle and late points in the movement, based on the 100 ms-wide windows shown in panels A and C. Asterisks represent significant differences in force between pFF and vFF responses at (*p* < 0.01, two-tailed t-test). Error bars show SEM.
(EPS)
